# Biodegradable Polymeric Nanoparticle-Based Drug Delivery Systems: Comprehensive Overview, Perspectives and Challenges

**DOI:** 10.3390/polym16172536

**Published:** 2024-09-07

**Authors:** Małgorzata Geszke-Moritz, Michał Moritz

**Affiliations:** 1 Department of Pharmacognosy and Natural Medicines, Pomeranian Medical University in Szczecin, Plac Polskiego Czerwonego Krzyża 1, 71-251 Szczecin, Poland; 2 Department of Pharmaceutical Chemistry, Pomeranian Medical University in Szczecin, Plac Polskiego Czerwonego Krzyża 1, 71-251 Szczecin, Poland

**Keywords:** nanoparticles, polymers, biodegradability, biocompatibility, drug delivery systems

## Abstract

In the last few decades, there has been a growing interest in the use of biodegradable polymeric nanoparticles (BPNPs) as the carriers for various therapeutic agents in drug delivery systems. BPNPs have the potential to improve the efficacy of numerous active agents by facilitating targeted delivery to a desired site in the body. Biodegradable polymers are especially promising nanocarriers for therapeutic substances characterized by poor solubility, instability, rapid metabolism, and rapid system elimination. Such molecules can be efficiently encapsulated and subsequently released from nanoparticles, which greatly improves their stability and bioavailability. Biopolymers seem to be the most suitable candidates to be used as the nanocarriers in various delivery platforms, especially due to their biocompatibility and biodegradability. Other unique properties of the polymeric nanocarriers include low cost, flexibility, stability, minimal side effects, low toxicity, good entrapment potential, and long-term and controlled drug release. An overview summarizing the research results from the last years in the field of the successful fabrication of BPNPs loaded with various therapeutic agents is provided. The possible challenges involving nanoparticle stability under physiological conditions and the possibility of scaling up production while maintaining quality, as well as the future possibilities of employing BPNPs, are also reviewed.

## 1. Introduction

**Figure 1:**
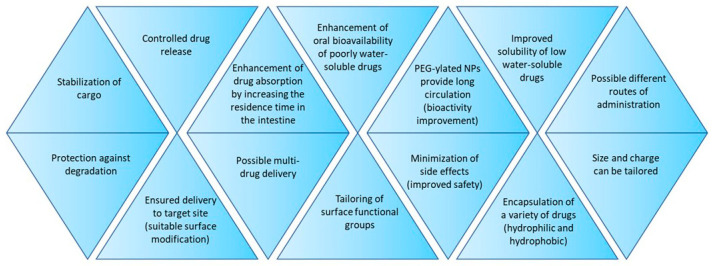
Advantages resulting from the usage of drug-loaded polymeric nanoparticles versus conventional (crystalline) active agents.

In the last decade, there has been a growing interest in the use of biodegradable polymeric nanoparticles (BPNPs) as drug carriers [1,2,3,4,5]. Polymeric carriers have many beneficial properties that improve the effectiveness of conventional drug therapies. Polymeric nanoparticles, like other nanomaterials used in biomedicine, have unique properties resulting from their nanometric size. These include an improved dissolution kinetics of encapsulated drugs that are mostly poorly or not at all soluble in water in crystalline form [1]. Drug molecules incorporated, encapsulated or adsorbed onto polymer matrices are better protected against degradation in physiological fluids [3]. Appropriate chemical modification of the surface of polymeric nanoparticles ensures controlled (slowed, accelerated) release of the drug at the target site [2]. The release of the drug can be initiated by various stimuli such as light, pH, magnetic field or temperature [3]. In addition, it is possible to attach ligands for receptors that are overexpressed, for example on the surface of cancer cells [6,7]. In this way, after delivering an as-prepared anticancer drug-loaded nanocarrier to the body, it is possible to direct the drug molecule to cancer cells and significantly reduce side effects for the entire body. Therefore, it seems that therapy with the use of nanoparticles is safer, as it allows one to reduce the dose of the drug delivered to the patient, ensures its local effect, and reduces the side effects of drugs that are usually characterized by significant toxicity [8]. The diversity of polymeric matrices makes it possible to use them as carriers for both hydrophilic and hydrophobic drugs [9]. The enormous progress made in recent years in the methods of polymer nanoparticle fabrication makes it possible to control their chemical composition, particle size and surface charge [2]. This, in turn, determines such features of the nanocarrier as biocompatibility, biodegradability, affinity for cells and distribution in the body. Not without significance for use as attractive drug carriers are also the excellent mechanical properties of most polymeric materials, including their flexibility and durability [2]. BPNPs are also effective drug carriers for oral administration [10]. In this case, they can provide protection against the breakdown of sensitive drugs in the harsh conditions of the gastrointestinal tract, as well as contributing to prolonging their residence in the intestines. These and other benefits of using polymeric nanoparticles as drug carriers are shown in Figure 1.

Despite the promising benefits of using polymeric nanoparticles as drug carriers, there are still many questions that need to be addressed further. The key issue seems to be the toxicity concerns [11,12]. A large number of publications describe modern and advanced methods for the preparation, functionalization and physicochemical characterization of polymeric nanoparticles. Far fewer research groups are involved in testing the cytotoxicity of these nanostructures in vitro, and even fewer are involved in in vivo studies on animal models. Another problem is to increase the scale of nanoparticle production while maintaining the repeatability of the formulation process [9]. The formulation and research of polymeric nanoparticles require in-depth and detailed research conducted by qualified research teams.

This review article summarized the latest publications devoted to the preparation, modification, physicochemical characterization, biological studies on and use of BPNPs as drug carriers. Particular attention is paid to the arrangement of information on polymeric carriers. Although many excellent reviews of BPNPs have been published, they usually describe a carefully selected topic of BPNPs, e.g., formulation, characterization or degradation. There are also reviews describing various aspects of polymeric nanoparticles but devoted to one type of polymeric matrix, such as gelatin, chitosan or PLGA. In this review article, the classification of polymer nanoparticles is clearly presented, their structure is described, and the method of placing the drug in the carrier, along with the advantages and disadvantages of nanoparticles based on natural and synthetic polymers, are listed. Then, the methods of formulating polymer nanoparticles are briefly described and the analytical techniques most commonly used for their characterization are described. After that, the biomedical applications of these carriers are presented, with an emphasis on drug delivery systems (DDSs). Possible pathways for the decomposition of polymeric nanoparticles are also described. A significant part of the work is devoted to describing the challenges that need to be met so that polymer carriers can be used in medicine while maintaining patient safety and achieving the effectiveness of therapy

## 2. Classification, Formulation, Characteristics, Bioapplications and Degradation of Polymeric Nanoparticles

### 2.1. Classification of Polymeric Nanoparticles

**Figure 2:**
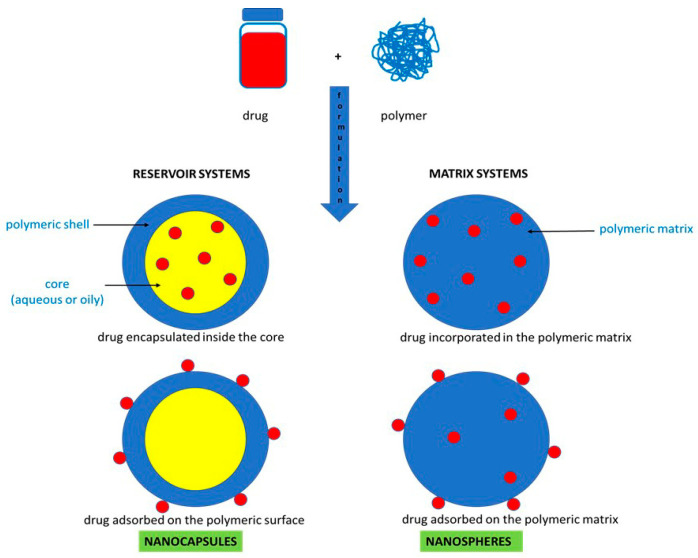
Types of polymeric nanoparticles according to the composition.

According to their composition, polymeric nanoparticles can be divided into nanocapsules and nanospheres [13,14]. Schematic representations of both structures are presented in Figure 2.

Nanocapsules are systems in which drug molecules are encapsulated in the nanoparticle core or are adsorbed on a polymeric shell. In nanospheres, the drug is encapsulated in the nanoparticle’s polymeric matrix or it is adsorbed on its surface. Polymer matrices used as drug carriers can be based on natural or synthetic polymers [1]. Among natural polymers, those derived from animals, plants, algae, fungi and bacteria can be distinguished [5]. All these matrices are characterized by excellent biocompatibility and biodegradability. In turn, the most popular biodegradable synthetic polymers used in the formulation of nanoparticles are polyglycolic acid (PGA), polylactic acid (PLA), poly(lactic-*co*-glycolic acid) (PLGA), poly-ε-caprolactone (PCL) and polyvinyl alcohol (PVA) [2,15]. The classification of polymer matrices and their most popular sources are presented in Table 1.

The most common and abundant natural polymers used in the formulation of nanoparticles are polysaccharides and polypeptides [14]. Some examples of polysaccharides used as drug carriers include chitosan [42], alginate [43], starch [44], cellulose [45], hyaluronic acid [46], and dextran [47]. To the polymeric matrices with a polypeptide structure belong collagen [48], gelatin [49], albumin [50], elastin [51], and silk fibroin [52]. Chitosan is a product of the deacetylation of chitin [1]. It is a product of animal origin. It does not dissolve in water, but it is soluble in weakly acidic solutions. Due to the presence of amine groups, chitosan possesses many benefits underlying its use as a drug carrier. These include mucoadhesion, in situ gelation and improved penetration through physiological barriers [1]. Alginate is soluble in water. It is a 1–4 linked α–L–guluronic acid and a β–D-mannuronic acid. This compound also exhibits excellent mucoadhesive properties. These properties are the result of the strong hydrogen bond formation between chitosan molecules and glycoproteins present in mucin through carboxyl–hydroxyl interactions. Starch is obtained from many plants such as potato, corn and rice [2]. It is made of amylose and amylopectin. Starch reveals excellent swelling and rheological properties, which make it an attractive drug carrier. One popular carrier of a drug with a protein structure is collagen. It is abundant in the human body. Collagen is found in cartilage and skin, among other places [53]. It exhibits gelling properties, can be sterilized, and is considered as a safe material for human beings. Gelatin is water-soluble protein obtained from collagen. It is a cheap and readily available polymer [9]. Gelatin has many beneficial properties grounding its application as the carrier in DDSs, such as stability or non-immunogenicity. However, due to its good solubility in water, gelatin binds the drug too loosely and requires prior chemical modification [3]. The advantage of gelatin is the possibility of modifying its isoelectric point and thus properly controlling the drug-binding process [3]. Albumin is a globular carrier protein. It is obtained from plants and animals and is isolated from human blood. This group of proteins represent attractive drug carriers due to their limited reactions in the immune system [53]. Silk fibroin is obtained from the cocoons of *Bombyx mori*. It exhibits very high mechanical strength. Additionally, it promotes cell proliferation and adhesion. It is an attractive candidate for use as a carrier in DDSs due to the controlled degradation rate [53]. Synthetic polymers used in the formulation of nanoparticles are obtained from various non-toxic monomers (e.g., natural metabolites such a lactic acid) by chemical synthesis. The degradation products of these carriers do not cause serious side effects to the body [2]. However, the biodegradation process of these polymer carriers is usually much longer compared to nanoparticles based on natural polymers. In comparison to the majority of BPNPs based on natural polymers, synthetic polymeric matrices are characterized by excellent mechanical properties. For this reason, polylactic acid is perfect for use as a carrier for controlled drug release [9]. A very popular synthetic polymer carrier with negligible toxicity is poly(lactic-co-glycolic acid). Poly–ε–aprolactone is a compound with a polyester structure and a hydrophobic nature. For this reason, it is successfully used as a carrier of hydrophobic drugs. Often, in order to obtain or enhance the desired properties such as mucoadhesion, hydrophilicity or faster degradation in the body, it is blended with other polymers [1]. The characteristics of natural and synthetic polymeric materials in regard to their application as the carriers in DDSs, divided into their advantages and disadvantages, are summarized in Table 2.

As indicated by the data presented in the table, both natural and synthetic polymeric matrices offer advantages and disadvantages in the context of their use as drug carriers. Both types of polymeric materials are biocompatible. Natural polymers are biodegradable and usually inexpensive. However, their structures are complex; they are often obtained in complex processes of extraction and are characterized by a lack of homogeneity [13]. Synthetic polymeric matrices are characterized by high stability and good chemical modification capabilities. Their production is repeatable, although sometimes expensive. Compared to natural polymers, these materials are characterized by higher toxicity and slower degradation [3].

### 2.2. Formulation of Polymeric Nanoparticles

Polymeric nanoparticles are formed from a polymer, a surfactant and an aqueous phase [1]. They can be formulated using various methods. The method of formulation of polymeric nanoparticles depends on both the properties of the polymer from which the nanoparticles are to be made and the properties of the drug to be placed in the polymeric matrix. The chosen method of formulation of polymeric nanoparticles should ensure their desired size and adequate drug load capacity. Contemporary methods of obtaining polymer nanoparticles are characterized by simplicity, safety and repeatability. The two main methods for obtaining polymer nanoparticles are self-assembly and emulsion methods [3]. The self-assembly method is based on the inter- and intramolecular interactions between the polymer molecules themselves and the drug molecules. In the emulsification method, nanoparticles are formed as droplets of one phase in the other phase. Typically, the polymer is dissolved in the drug-containing organic phase, then mixed with a surfactant and sonicated in the aqueous phase. Finally, the nanodroplets are formed. The emulsion is stirred until the solvent evaporates and leaves hard polymeric nanoparticles. The group of Shivakumar [61] distinguished three main methods for obtaining polymeric nanoparticles. The first is the method of obtaining nanoparticles from dispersions of preformed polymer. It is a popular method of obtaining BPNPs based on synthetic polymeric matrices such as PLA and PLGA [1]. This method includes solvent evaporation, nanoprecipitation and emulsification/solvent diffusion. The second method is the preparation of nanoparticles from polymerization of monomers. This method includes emulsion, microemulsion and interfacial polymerization. The last method to obtain polymeric nanoparticles is ionic gelation and coacervation. In this method, nanoparticles are often prepared from natural hydrophilic polymers such as gelatin, chitosan or alginate. The most popular methods of obtaining polymeric nanoparticles are presented in Table 3.

The polymer nanoparticles obtained by any method can undergo a modification process in order to improve properties relevant to their applications as drug carriers. This can be a chemical, physical or ultrasonically assisted process. The modification process is aimed at improving the stability of the system, preventing the aggregation of nanoparticles or protecting them from alterations [53].

### 2.3. Characteristics of Polymeric Nanoparticles

The in-depth physicochemical characterization of the nanomaterial is required for its eventual use as a carrier in DDSs. To date, no procedures or regulations have been developed for the characterization of nanoparticles for biomedical applications [9]. However, it is obvious that the behavior of nanoparticles in the body results from the nanocarrier’s physicochemical properties. Before being used as a drug carrier, polymeric nanoparticles must be subjected to deep physicochemical characterization as well as biological studies. Toxicity studies are of particular importance. The most relevant methods used for the physicochemical characterization of polymeric nanoparticles are summarized in Table 4.

The analytical techniques employed to determine the size and morphology of nanoparticles are electron microscopy techniques and DLS analysis. In the SEM imaging method, information about the nanoparticle’s morphology and surface is obtained [87]. In TEM analysis, a two-dimensional image is obtained. Using this microscopic technique, it is possible to determine, among other things, the thickness of the nanocapsule polymeric shell [88]. The AFM method gives the opportunity to study the particle surfaces with nanometer resolution [89]. DLS analysis is used to estimate the sizes of nanoparticles in solution [90]. The analytical techniques used to determine the surface properties and stability of the obtained polymeric nanocarriers are Raman spectroscopy, XPS and FT-IR, among others. These methods allow us to determine the chemical composition of the nanoparticle surface and obtain information about the functional groups on the surface of the nanoparticles [91]. DSC is used to determine the possible interactions between a polymeric matrix and the drug placed in it. Additionally, this method allows one to obtain information about the physichochemical state of the polymer. Phase transition such as glass transition, melting and crystallization can be detected using the DSC method [91].

### 2.4. Bioapplications of Polymeric Nanoparticles

**Figure 3:**
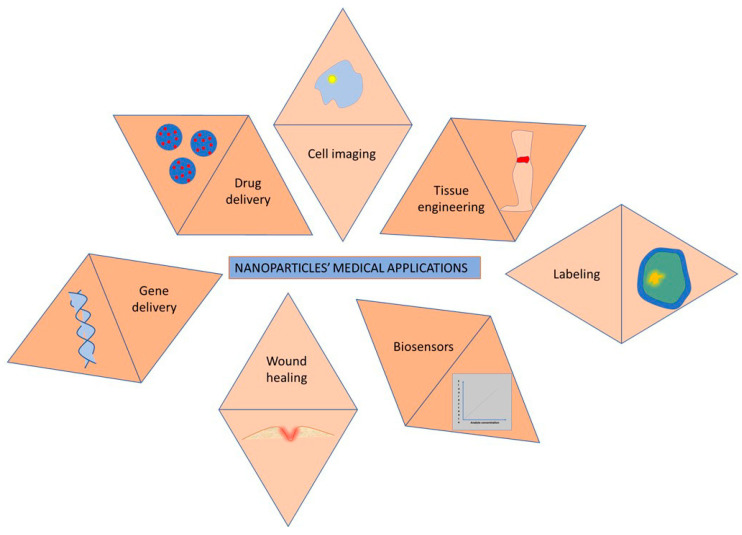
Medical applications of nanoparticles.

Nanomaterials have found many applications in the field of medicine. Thus polymer nanoparticles are used, among other applications, in tissue regeneration [92], gene delivery [93], drug delivery [94], wound healing [95], biosensing [96], labeling [97] and bioimaging [98], as schematically presented in Figure 3.

**Figure 4:**
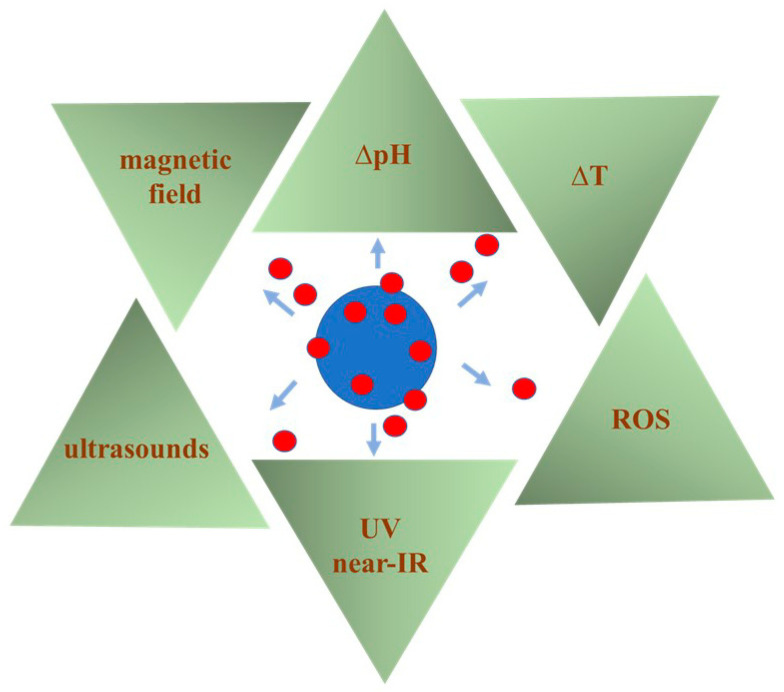
Stimuli causing the release of encapsulated/adsorbed drug from polymeric nanoparticles (ROS—reactive oxygen species, ∆T—temperature change).

One of the very frequently studied uses of BPNPs is their application as carriers in DDSs. Nanomaterials provide exciting properties for use in more effective therapies. BPNPs can be used as carriers of a wide range of active substances such as antibiotics [99], antiviral drugs [100], antifungal drugs [101], anticancer drugs [102], anti-inflammatory drugs [103], drugs used in eye diseases [104], active compounds of natural origin such as antioxidants [105], essential oils [106] and proteins [107]. By properly controlling the formulation process and eventual chemical modification of polymeric nanoparticles, it is possible to achieve controlled and targeted drug delivery in the body. An active substance can be released after administration into the body under the influence of various factors, such as the specific pH at the malignant tissue, magnetic field, or temperature, as is schematically presented in Figure 4.

Polymeric nanoparticles are an attractive drug carrier for drugs used in eye diseases. The group of Pujol [8] decribed the preparation of dexibuprofen-loaded BPNPs to be used in ocular inflammation. PLGA was employed as a matrix to encapulate this poorly water-soluble anti-inflammatory drug. As the bioavailability of drugs in ocular tissues is very low, the usage of polymeric nanocarriers instead of traditional eye-drops markedly improved the anti-inflammatory effect of dexibuprofen in New Zeland albino rabbits, simultaneously reducing the side effects in whole organism. Shinde et al. [108] loaded dorzolamide in chitosan nanoparticles functionalized with 6-O-carboxymethyl groups. The ocular irritation potential of the prepared formulation was examined via a reduction in intraocular pressure in normotensive rabbits. Increased dorzolamide entrapment efficiency was noted for 6-O-carboxymethyl-funtionalized nanoparticles as compared to the non-modified nanocarrier. In vivo experiments revealed the prolonged antiglaucoma effect of functionalized chitosan nanoparticles as compared to the non-modifed nanocarrier. The group of Sakhi [109] prepared moxiflocacin hydrochloride-loaded PLGA nanoparticles with improved residence time, and hence increased antibiotic absorption from the corneal surface. The prepared formulation revealed a high initial drug release rate for 6 h followed by its sustained release. The in vivo experiments performed on a rabbit model confirmed no irritation effect to the eye. The group of Alqurshi [110] described the ocular anti-inflammatory activity of chitosan-deoxycholate nanoparticles loaded with prednisolone. A two-fold increase in drug release in a simulated tears fluid was observed when incorporated into the nanoparticle gel formulation. In vivo experiments on a female Guinea pig model revealed improved anti-inflammatory effects of the prepared nanoformulation as compared to the micronized drug-loaded gel.

The delivery of anti-cancer drugs using polymer carriers is also widely studied by various research groups. Xiong et al. [72] reported on the preparation of paclitaxel- and curcumin-loaded BPNPs employing the tri-block copolymer poly(ε-caprolactone)-poly(ethylene glycol)-poly(ε-caprolactone) as a polymeric matrix. The in vitro and in vivo antitumor effects of the prepared multi-drug nanocarrier against breast cancer were examined. It was found that polymeric nanoparticles were more readily uptaken by tumor cells in vitro. After intravenous administration to a BALB/c nude mouse, a significant inhibition of tumor growth with reduced side effects as compared to free drugs was observed. The group of Shavandi [111] formulated 5-fluorouracil-loaded PLGA nanoparticles for colorectal cancer therapy. The drug release experiments revealed that 5-fluorouracil encapsulation provided more controllable release as compared to the free drug. The results of DAPI staining and flow cytometry indicate that the prepared formulation was able to kill cancerous colon cells at a gradual rate and safe drug dosage. The group of Fahmy [112] prepared polymeric nanoparticles loaded with essential oil extracted from Boswelia sacra. The polymeric matrix consisted of blended PLGA and PCL. The results of the in vitro cytotoxicity MTT test reveal that Boswelia sacra oil entrapped in polymeric nanoparticles improved the anti-breast cancer effect via enhancing apoptosis as compared to the control and free essential oil. Ji and co-workers [6] prepared chitosan-coated PLGA nanoparticles loaded with carboplatin and reported enhanced antiproliferative effects in cervical cancer cells of an as-prepared formulation. In order to provide targeted delivery to malignant tissues, nanoparticles were decorated with folic acid. Folic acid is a ligand for folate receptors that are overexpressed on the surfaces of cancer cells. Nanoparticles exhibited enhanced affinity to cancer cells and showed superior cytotoxicity action as compared to non-functionalized nanocarriers. It was found that the chitosan layer played a protective role and controlled the carboplatin release rate from the formulation. The group of Khan [113] reported on the preparation of PLGA nanoparticles co-loaded with rapamycin (drug used in the therapy of breast cancer) and piperine (chemosensitizer). In vitro experiments revealed sustained drug release for weeks. Moreover, the uptake of rapamycin was increased in the presence of piperine. The results of pharmacokinetic studies exhibited the better absorption profile of drugs from a polymeric nanocarrier as compared to drug suspension. It was found that the co-delivery of both active agents is a promising approach for the treatment of breast cancer.

Polymeric nanoparticles have also been studied as carriers of antifungal, antiviral, antileishmaniasis, and many other drugs. Gamil at al. [114] prepared miconazole-loaded chitosan nanoparticles gels to be administered in diabetic patients with oral candidiasis in randomized control clinical trial. The prepared nanocarrier loaded with an antifungal drug was effective in controlling oral candidiasis symptoms and reducing Candida colonization. It was suggested that miconazole-loaded chitosan nanoparticles exhibited higher affinity to fungal cells as compared to healthy cells. Dahmane and co-workers [115] described a formulation of zidovudine-loaded chitosan nanoparticles. It was found that the encapsulation ability of this anti-human immunodeficiency virus (HIV) drug and its release profile were strongly affected by the inherent properties of chitosan, such as its molecular weight and by the preparation conditions. The results of in vitro experiments confirm the continuous slow drug release for ca. 20 h. Khan and co-workers [116] prepared miltefosine-loaded chitosan nanoparticles for the treatment of cutaneous leishmaniasis. The results of the in vitro cytotoxicity MTT assay reveal the antileishmanial effects of the prepared formulation on promastigotes. In vivo experiments on infected BALB/c mice proved that after the oral and intralesional injection of the drug-loaded nanocarrier, the lesions were significantly healed. Tzeyung et al. [117] formulated chitosan nanoparticles loaded with rotigotine for nose-to-brain delivery. In vitro release studies demonstrated sustained drug release from the nanocarrier. The prepared nanoformulation revealed improved ex vivo nasal permeation when compared with rotigotine solution. Histopathological experiments proved no toxicity or structural damage of nasal mucosa. The performed experiments indicate the possibility of using chitosan nanoparticles as the carrier of rotigotine. The nanoformulation administered via nose-to-brain route can be used as an alternative to conventional therapies of Parkinson’s disease. Gupta and co-workers [118] revealed the enhanced hepatoprotective activity of silymarin on Albino mice when loaded in chitosan nanoparticles. In vitro dissolution experiments indicated the sustained drug release from the prepared nanocarrier. It was found that nanoparticle size, drug entrapment efficiency and drug release rate strongly depend on various formulation variables. Bhokare et al. [119] prepared chitosan nanoparticles loaded with rosuvastatin antihyperlipidemic drug. In vitro experiments revealed sustained drug release up to 10 h. It was found that drug release was affected by the rosuvastatin to chitosan ratio and the nanoparticle size. Other examples of the use of polymeric biodegradable materials as carriers for drugs from different therapeutic groups are presented in Table 5.

### 2.5. Degradation of Polymeric Nanoparticles

**Figure 5:**
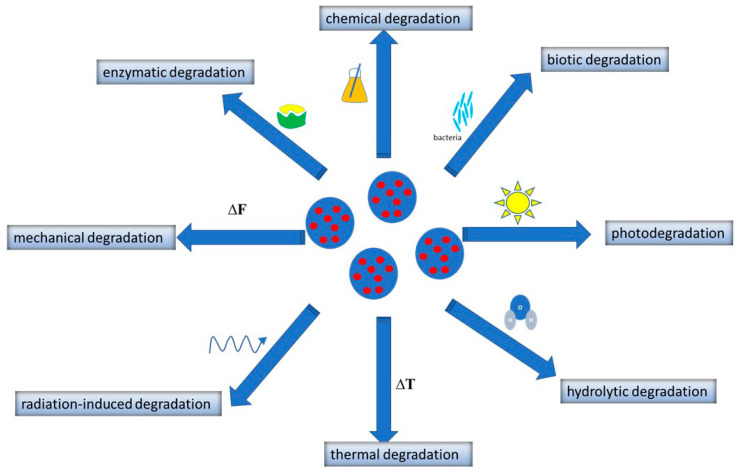
Possible mechanisms of polymeric nanoparticle degradation.

Biodegradation is the process in which biocompatible or harmless by-products are formed [140]. Biodegradable polymers are high-molecular-weight compounds that degrade over time in the presence of physiological fluids [53]. The factors that affect the rate and course of polymer degradation are its chemical composition, structure, molecular weight, polydispersity and distribution of monomers [141]. The biodegradation of natural polymers (polysaccharides, polypeptides) occurs through biological processes such as hydrolysis [2]. Protein and polysaccharide nanoparticles are biodegradable, as in the process of their degradation no harmful by-products are formed. The majority of natural polymers are broken down with the participation of enzymes. For example, polysaccharide matrices are enzymatically degraded in the human body by lysozymes and amylases [2]. It is worth noting that the biodegradation process of these polymer matrices can be controlled. For example, in the case of chitosan, it can be achieved via varying molecular weight, the degree of deacetylation and chemical modification [3]. In this way, a specific drug release profile in the body can be achieved. Biodegradable synthetic polymers are mainly broken down by the hydrolysis of ester bonds [53]. It was reported that polymers with polar groups decompose faster compared to those with non-polar functions. Although degradable synthetic polymers are broken down over time by metabolic processes, they may reside in the circulatory system for some time and can accumulate in organs such as lung, liver and spleen [4]. As an example it was shown that the degradation of PCL lasts about 2–3 years [53]. There are also synthetic polymers such as PLA and PLGA that decompose quite quickly into completely safe by-products. PLA is biodegradable by hydrolysis. Water molecules break the ester bond in the PLA backbone, leading to the formation of lactic acid [1]. This by-product is easily metabolized in the body or eliminated in the urine, and has no harmful effects on the body. PLGA is broken down by the hydrolysis of ester bonds in a water environment to lactic acid and glycolic acid. Both of these constitutive monomers are naturally present in the body, and under physiological conditions participate in various metabolic pathways [59]. Polymer degradation can occur under the influence of various factors such as radiation, moisture, heat, magnetic field, mechanical force, and biological and chemical factors [141], as is schematically presented in Figure 5.

Under the influence of these factors, changes occur in the polymeric composition, molecular weight, and chemical and structural properties. Depending on the factor responsible for degradation, hydrolytic, photochemical, oxidative, thermal, radiation-induced or microbial decomposition can be distinguished [141]. Usually, many degradation mechanisms occur simultaneously, so it is a difficult process to predict.

## 3. Perspectives and Challenges

**Figure 6:**
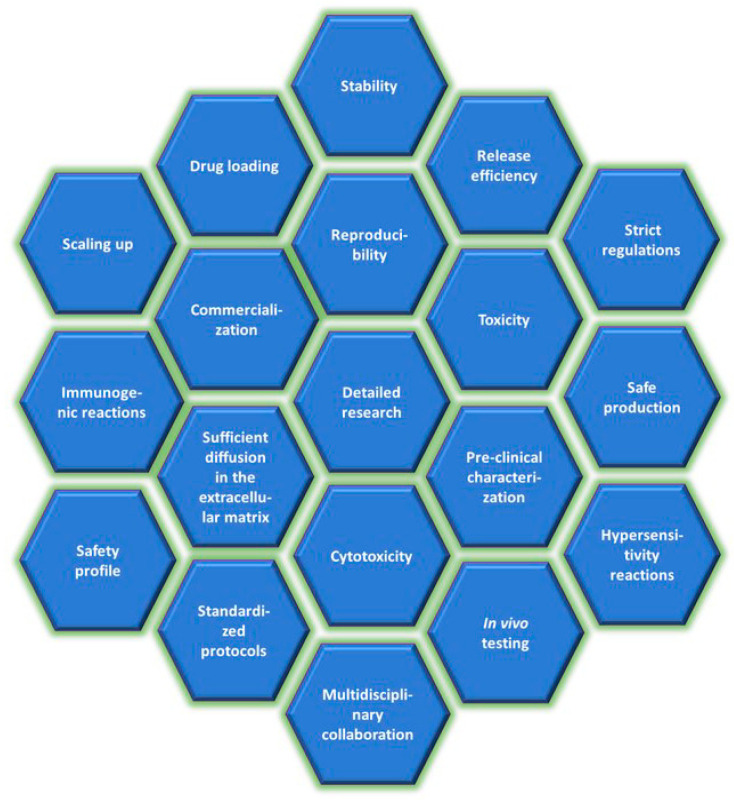
Challenges in the biomedical application of polymeric nanoparticles.

There is an increasing interest in the application of BPNPs in nanomedicine. They offer great potential utility as the carriers in DDSs [1,9,13,59]. Polymeric nanoparticles can be used to deliver many types of drugs [47,66,77,103,112,119]. Multi-drug delivery is also possible [72,113,125]. Additionally, drug release can be triggered by numerous stimuli [66,126]. This allows for stimulus-dependent controlled and targeted release, the minimization of side effects and the improvement of therapeutic efficacy. Biodegradable polymers are very attractive drug carriers with merits of biocompatibility and biodegradability [2,3,4,5,9,54]. They seem to be easily eliminated from the body, which makes them excellent candidates to be used as the matrices in nanoformulations [2,3,142]. However, before the successful clinical application of BPNPs, several issues need to be addressed. The successful clinical usage of BPNPs is limited by various drawbacks such as changes in the nanoparticle physicochemical properties (nanoparticle size, aggregation, surface charge), premature drug release, insufficient drug encapsulation efficiency, problems with achieving the desired drug release profile, and improper distribution in the body [1,2,9]. The challenges for researchers working on pharmaceutical formulations based on BPNPs are shown in Figure 6.

A significant challenge is to ensure the stability of the nanoformulations being prepared. Nanoparticles cannot aggregate, and degrade too early or too late in the body. Furthermore, they must not prematurely release the incorporated drug. It is also necessary to pay attention to the appropriate storage conditions of nanoformulations, such as humidity, temperature or exposure to light. It seems that the commercial use of nanoparticles brings some risk for the patient. Particle nanometric size results in the increased reactivity and toxicity of the carrier [1]. This is due to the fact that small particles can penetrate biological membranes and reach various tissues without being recognized by the reticuloendothelial system [15]. Even for biodegradable materials, safety assessment remains one of the top priorities for nanomedicine. Even if the material is biodegradable, it does not mean that it will not have any side effects after administration to such a complex system as the human body. Chemical modification of BPNPs or the incorporation of certain drugs may cause some toxicity concerns. The toxicity of nanoformulations may vary depending on the dose and duration of exposure. Polymeric nanoparticles can generate reactive oxygen species. The reactions from the immune system and overproduction of inflammatory mediators are also likely [54]. The effectiveness of the therapy is greatly influenced by the amount of drug loaded in the nanoparticle and its appropriate release profile. This challenge seems to be particularly important in the case of hydrophobic drugs and those that require the administration of high doses. The release profile of the drug depends on physiological conditions at the site of application, such as pH or the presence of enzymes, which are difficult to control. Finding the right routes of BPNPs administration is challenging due to limitations such as the poor water-solubility of drugs, and their low bioavailability and instability. The transfer of the results of basic research into clinical application is very challenging. It should be noted that the cost of producing nanoparticles is also quite high. Another challenge is the reproducibility of the nanoparticle fabrication process [9]. The process of producing nanoparticles on a large scale and for clinical applications must be reliable and cost-effective, simultaneously maintaining the quality of the resulting formulations, including homogeneous nanoparticle size, appropriate drug loading and satisfying stability. The large-scale production of nanoparticles and ensuring the same quality of all batches is still challenging. There are problems with preparing nanoparticles of homogeneous size and shape. Moreover, initial burst release effects or incomplete drug release are possible. To date, most scientific publications describe only the in vitro characteristics of BPNPs. In vivo studies and clinical trials are very rare. Despite the huge progress observed in the field of BPNP synthesis, more research should be devoted to in vivo studies, interactions with human cells and long-term stability in biological fluids. The elimination of nanoparticles from the body is also an important issue. Drug delivery employing polymeric matrices as the drug carriers offers excellent opportunities. Possibly, novel protocols will provide BPNPs with enhanced drug encapsulation capacity, ensuring satisfying stability and effective delivery through complex biological barriers. We think that novel nanoformulations will be safe, more specific, and programmed, and will simplify existing therapies. We hope that nanoparticle-based therapies will be more affordable and easier to use. In order to achieve this goal, more research should be carried out in multidisciplinary research groups consisting of biologists, chemists, engineers, doctors, physicists and specialists in the field of nanotechnology. The challenge is considerable, but the benefits for the patient seem to be a promising prospect.

## Figures and Tables

**Table 1:** Types of natural and synthetic polymers used in the formulation of biodegradable polymeric nanoparticles.

Type of Polymeric Nanoparticles	Origin	Polymeric Matrix	Ref.
Natural	Algae	Alginate	[16]
		Carageenan	[17]
		Fucoidan	[18]
	Animals	Albumin	[19]
		Casein	[20]
		Chitosan	[21]
		Collagen	[22]
		Gelatin	[23]
		Keratin	[24]
		Silk fibroin	[25]
	Bacteria	Dextran	[26]
		Gellan gum	[27]
		Levan	[28]
		Xanthan gum	[29]
	Fungi	Pullulan	[30]
	Plants	Cellulose	[31]
		Gliadin	[32]
		Guar gum	[33]
		Pectin	[34]
		Starch	[35]
		Zein	[36]
Synthetic	---	Poly-ε-caprolactone (PCL)	[37]
		Polyglycolic acid (PGA)	[38]
		Polylactic acid (PLA)	[39]
		Poly(lactic-*co*-glycolic acid) (PLGA)	[40]
		Polyvinyl alcohol (PVA)	[41]

**Table 2:** Characteristics of polymeric materials used in the process of nanoparticle formulation.

Polymer Type/Chemical Composition		Polymeric Material	Advantages	Disadvantages	Ref.
**Natural**	Polysaccharides	Alginate	Water-solubility Biodegradability Biocompatibility Mucoadhesion Gel-forming capability Low immunogenicity Low cost Non-toxicity	Low mechanical properties Sterilization is difficult	[2,5,54,55,56]
		Carageenan	Forms highly viscous solutions or elastic gels Protein-binding properties Emulsion stabilizer	Low gel strength Anti-coagulant properties	[53,55]
		Cellulose	Abundant in nature Biocompatibility Low toxicity Low cost	Insolubility in many common solvents (difficult processing) Lack of flexibility Lack of thermoplasticity	[55]
		Chitosan	Mucoadhesion In situ gelation Biocompatibility Anti-bacterial properties Biodegradability	High in vivo degradation rate Low mechanical strength Hard to control NP size Low flexibility Not easy to process Insoluble in neutral solutions (dissolves in diluted acidic solutions)	[2,55]
		Chitin	Abundant in nature Biodegradability Biocompatibility Non-toxicity Mucoadhesive Easy chemical modification	The content of impurities depends on the chitin source and preparation method Poor solubility at physiological pH	[53]
		Dextran	Biocompatibility Anti-thrombotic properties Good water solubility Easy functionalization Biodegradability Good rheological and thermal properties	High cost Non-available Encapsulated drugs are released very fast	[53,55]
		Fucoidan	Non-toxicity Biodegradability Biocompatibility Certain biological properties (anti-oxidant, anti-inflammatory, anticoagulant)	The quality of fucoidan depends on the species from which it is extracted	[53]
		Hyaluronic acid	Easy chemical modification Interacts with cells (cell proliferation, angiogenesis, matrix organization)	Absorbs large amount of water Rapid degradation Brittle High cost Poor mechanical properties	[53,54]
		Pullulan	Biodegradability Biocompatibility Non-toxicity Water-solubility Forms stable, viscous non-hygroscopic solutions Adhesion properties Non-immunogenicity	Highly expensive Brittle Low mechanical strength	[55]
		Starch	Biodegradability Low cost Biocompatibility Easily available Swelling properties	Very high viscosity Low mechanical properties Fragile (very high water uptake) Lack of flexibility Brittle Degrades before its melting temperature Difficult processability	[2,55]
	Proteins	Albumin	Biodegradability Non-toxicity Highly abundant Biocompatibility Non-cytotoxicity Water-solubility	Possible immunogenic reactions Expensive	[2,55]
		Collagen	Low immunogenicity Excellent cell adhesion Biocompatibility Biodegradability Abundant in human body	Low mechanical strength Variability depending on collagen source	[2,55]
		Elastin	Abundant in human body It can retain its original shape even after stretching Ability to self-assemble (response to various temperatures)	Not always biocompatible Difficult to alter	[53]
		Gelatin	Biocompatibility Biodegradability Thermo-reversible gelation properties Ability to form hydrogels Eco-friendly Low cost Water-solubility Easily available Great stability Non-immunogenic The isoelectric point can be modified to optimize the loading of charged drugs	Fast degradation in physiological fluids Brittle	[2,9,53]
		Silk fibroin	Biocompatibility Good elastic properties Very high mechanical strength Controlled degradation rate	Degradation with immunogenic reactions	[2,53]
Synthetic		Poly-ε-caprolactone (PCL)	Biocompatibility Non-toxicity Good mechanical properties Flexibility Slow biodegradation (useful in controlled drug release) Good rheological properties	Low bioactivity Hydrophobicity (poor cellular adhesion) Use of toxic solvents during synthesis	[53,54,57]
		Polyglycolic acid (PGA)	Excellent mechanical properties Long-term stability Soft Biodegradability High melting point	Short biocompatibility (in contact with biological fluids) Hydrophobicity (difficulties in interaction with cells) Insolubility in many common solvents Rapid degradation rate	[58]
		Polylactic acid (PLA)	Biodegradability Biocompatibility High mechanical strength Eco-friendly	No cell adhesion Expensive Chemically inert Poor stability in heat Very brittle	[3,55]
		Poly(lactic-*co*-glycolic acid) (PLGA)	Biodegradability Biocompatibility High stability Low toxicity	Before degradation the polymer remains in the circulation and then accumulates in the main organs like liver, lung, and spleen	[3,4,54,59,60]
		Polivinyl alcohol (PVA)	Biocompatibility Water-solubility Flexibility Low cost	Very high water uptake No cell adhesion	[2]

**Table 3:** Techniques used during polymeric nanoparticle preparation.

Technique	Type of Polymeric Nanoparticles.	Loaded Drug	Ref.
Desolvation	Zein	---	[62]
Emulsification solvent evaporation	Poly(caprolactone) (PCL), poly(lactic acid) (PLA), poly(lactide-co-glycolic acid) (PLGA)	Coumarin-6	[63]
	Poly(lactide-co-glycolic acid) (PLGA	Sparfloxacin, tacrolimus	[64]
Emulsion polymerization	Poly(*ε*-caprolactone) (PCL), poly(vinylpyrrolidone) (PNVP)	Cisplatin	[65]
Microfluidization	Shellac	Curcumin	[66]
Ionic gelation	Chitosan	Budenoside	[67]
	Chitosan	Curcumin	[68]
Nanoprecipitation	Poly(ethylene glycol) (PEG)-block-poly(lactic-co-glycolic acid) (PLGA)	Ketamine	[69]
	Polylactic acid (PLA), poly(lactic-co-glycolic acid) (PLGA)	Ferulic acid	[70]
Polyelectrolyte complexation	Gellan gum, chitosan	---	[71]
Self-assembly	Tri-block copolymer poly (ε-caprolactone)-poly(ethylene glycol)-poly(ε-caprolactone) (PCL-PEG-PCL, PCEC)	Paclitaxel, curcumin	[72]
Spray-drying	Bovine serum albumin (BSA)	Rutin	[73]

**Table 4:** Analytical techniques used for characterization of polymeric nanoparticles.

Technique	Knowledge Obtained	Ref.
Atomic force microscopy (AFM)	Surface texture Roughness Particle size distribution Aggregation	[74]
Cryo-transmission electron microscopy (cryo-TEM) High-resolution transmission electron microscopy (HR-TEM) Transmission electron microscopy (TEM)	Structure Size Size distribution Shape heterogeneity Aggregation	[74,75,76]
Differential scanning colorimetry (DSC)	Drug–polymer interaction Physicochemical state	[74,77]
Dynamic light scattering (DLS) Photon correlation spectroscopy (PCS)	Size Shape Polydispersity Surface charge	[12,78,79]
Fluorimetry High-performance liquid chromatography (HPLC) UV-Vis spectrophotometry	Drug content In vitro drug release	[78,80]
Fourier-transform infrared spectroscopy (FT-IR) Raman spectroscopy	Chemical composition Functional groups	[74,77]
Mass spectrometry (MS)	Molecular weight Composition Structure Surface properties	[81]
Nuclear magnetic resonance (NMR)	Chemical composition Structure Purity	[82]
Scanning electron microscopy (SEM) Scanning tunneling microscopy (STM)	Geometry Topography Composition Size Size distribution Aggregation	[74,80,83]
Small-angle neutron scattering (SANS) Small-angle X-ray diffraction (SAXS)	Shape Core/shell morphology	[84,85]
X-ray photoelectron spectroscopy (XPS)	Elemental and chemical composition at the surface	[86]

**Table 5:** Drugs encapsulated in polymeric nanoparticles.

Group of Drugs	Encapsulated Active Agent	Polymeric Matrix	Ref.
Antibiotics	Ciprofloxacin Gentamycin Tetracycline	Chitosan	[120]
	Polymyxin B	Hyaluronic acid Poly(lactic-*co*-glycolic acid)	[121]
	Spectinomycin and chloramphenicol	Gelatin	[122]
	Vancomycin	Soy protein	[123]
Anticancers	Carboplatin	Poly(lactic-*co*-glycolic acid)	[124]
	Docetaxel Gemcitabine	Albumin	[125]
	Doxorubicin siRNA	Starch	[7]
Antidiabetics	Insulin	Dextran	[126]
	Metformin	Alginate	[127]
Antifungals	Amphotericin A	Gelatin	[128]
Anti-inflammatory drugs	Ibuprofen	Starch	[129]
Antioxidants	Berberine	Polylactic acid	[130]
	Curcumin	Chitosan	[131]
	Geranyl cinnamate	Poly-ε-caprolactone	[132]
	Quercetin	Chitosan	[133]
	Resveratrol	Albumin	[134]
Antivirals	Stavudine	Chitosan	[135]
Essential oils	Basil essential oil	Chitosan	[136]
	Green tea essential oil Peppermint essential oil	Chitosan	[137]
	Oregano essential oil	Alginate Chitosan	[138]
Hormones	17β-Estradiol hemihydrate	Collagen	[139]

## Data Availability

Not applicable.
